# Unmasking a health care system: the Dutch policy response to the Covid-19 crisis

**DOI:** 10.1017/S1744133121000128

**Published:** 2021-03-05

**Authors:** Iris Wallenburg, Jan-Kees Helderman, Patrick Jeurissen, Roland Bal

**Affiliations:** 1 Erasmus School of Health Policy & Management, Erasmus University Rotterdam, Rotterdam, the Netherlands; 2 Radboud University, Nijmegen, the Netherlands; 3 Radboud UMC, Nijmegen, the Netherlands

**Keywords:** Covid-crisis, Netherlands, health care system resilience

## Abstract

The Covid-19 pandemic has put policy systems to the test. In this paper, we unmask the institutionalized resilience of the Dutch health care system to pandemic crisis. Building on logics of crisis decision-making and on the notion of ‘tact’, we reveal how the Dutch government initially succeeded in orchestrating collective action through aligning public health purposes and installing socio-economic policies to soften societal impact. However, when the crisis evolved into a more enduring one, a more contested policy arena emerged in which decision-makers had a hard time composing and defending a united decision-making strategy. Measures have become increasingly debated on all policy levels as well as among experts, and conflicts are widely covered in the Dutch media. With the 2021 elections ahead, this means an additional test of the resilience of the Dutch socio-political and health care systems.

## 1. Introduction

The Covid-19 pandemic has put policy systems to the test. The acute crisis of the virus outbreak in the late winter of 2020, which resulted in an overflow of severely ill patients in hospitals suffering from yet unknown illnesses, has evolved into a more enduring crisis that challenges the sustainability of national political-economic and health care systems (Bal *et al*., 2020; Boin *et al*., 2020*a*). In the Netherlands, the virus outbreak has moved the state in the centre of socio-political regulations and health care decision-making. This contrasts with the shift to decentralization and regulated competition that has characterized Dutch health care policies in the past three decades. Although public health implies a centralized approach, the decentralized and competitive governance logic of the Dutch health care system evokes divergent interests. This layered and fragmented institutional infrastructure, we argue in this paper, has put the central government in a difficult position to stipulate stringent policy measures to control the virus outbreak. We show how the government first more or less succeeded in the creation of collective action through ‘tactfully’ aligning public and private interests, supported by the felt urgency and willingness of a wide range of stakeholders – including the general public. Yet, we show, the government gradually lost control when the crisis became more enduring and conflicting interests and ideologies became more apparent while regional actors failed to pick up their regulating role, arguing for a more decisive and central authority. We use logics of decision-making during crisis (Allison, 1971; March and Olsen, 1989; March, 1994) and tact (Kornberger *et al*., 2019) to illuminate how collective action has been orchestrated and challenged in the unfolding Covid-crisis. In doing so, we aim to ‘unmask’ the institutionalized resilience-capacity of the Dutch health care system.

A crisis is commonly described as an unanticipated, surprising and ambiguous event posing a significant threat and leaving only a short decision time (Kornberger *et al*., 2019). Following the study of Colin Hay, crises are not simply a condensation of contradictions that produce a condition of rupture and breakdown. For an event to turn into a crisis, it must be perceived as such by agents capable of making a decisive intervention at the level at which the crisis is identified (Hay, 1996: 254). Which actors can take up this role, how ‘decisive’ the intervention is and what it comprehends, depends to a large extent on the institutional features of a policy system (Ansell *et al*., 2016). In this paper, we distinguish four different logics of decision-making. March and Olsen (1989) distinguish two such logics: the logic of consequence and the logic of appropriateness. The logic of consequence reflects a rational choice paradigm based on ‘a product of mind and choice’ rather than role, routine, intuition or emotion. It supposes the presence of calculated choice between alternatives. The logic of appropriateness, on the contrary, underscores decision makers' ability to read, and respond to ‘cues and prompts’ based on shared repertoires and institutionalized roles (March, 2006: 203). It relies on elements of tradition, rules, routines, identity and emotion, which is both deliberate and future-oriented. Building on these two logics, Kornberger *et al*. (2019) point out the logic of ‘tact’. Drawing on an empirical analysis of the refugee crisis in Vienna, they argue that collective actors that do not have shared histories may collectively orchestrate decisions through a way of ‘feeling out’; getting a sense of what is going on and switching between cognition and action. The logic of tact stresses the critical interplay of thought and action in decision-making under pressure (p. 260). In the ‘Essence of Decision’, Graham Allison distinguishes a fourth alternative logic of decision-making: the logic of bureaucratic/governmental political decision-making in which state actors seek to achieve separate goals, which may conflict with each other. Within this logic, individual decision-makers represent various and multiple – potentially conflicting – public interests: where they sit determines where they stand (Allison, 1971).

In this paper, we examine how policy decision-making happened in the unfolding Covid-crisis in the Netherlands, building on these four logics. We start with an overview of how the crisis manifested itself in the Netherlands, and how the machinery of policymaking unfolded in the course of the year. We set the scene by pointing out specific moments and debates that stood out in policy decision-making. We then describe the institutional setting of the Dutch health care system, and how the Covid-crisis plays out in the midst of (diffuse) legal requirements and both traditional and new institutionalized arrangements of governing health care and public health. In the empirical sections that follow, we deepen the analysis through the discussion of three topics that were key to crisis decision-making in the Netherlands: the alignment of hospital care; the negotiation of scarcity and the contested role of experts and expertise in policy-making.

## 2. Captured by a new virus^1^

The Netherlands more or less followed the pace of the Covid-19 outbreak in other European countries. The first wave started late Winter 2020, followed by a second one in September which, after a brief decline, worsened in the course of December. The first Covid-patient (‘Patient Zero’) was confirmed at the end of February during a live talk show in which the Minister of Health answered questions about the virus outbreak in Europe. The virus was brought in by people returning from skiing trips in Northern-Italy and Austria. Carnival parties in the Southern regions of the country (where carnival is traditionally celebrated) induced a quick spread of the virus. The outbreak first restricted to the South, but mid-March also people in other parts of the country turned sick. Hospitals soon got overwhelmed with Covid-patients, particularly in the most contaminated regions. In those first weeks, the crisis (and policy-making) was framed by scarcity: there was a lack of personal protection equipment (e.g. high-quality face masks and gloves), specialized nurses, intensive care unit (ICU)-beds and ventilators (Wallenburg *et al*., 2020; Boin *et al*., 2020*b*). Late March, the possibility of a ‘Code Black’ was high on the policy agenda as ICU capacity threatened to reach its limits, forcing politicians and physicians to make choices between life and death. Yet, this stage was never reached.

Policy-making was largely framed by the very acute hospital crisis – also drawing attention away from nursing homes and mental care (Kruse *et al*., 2020). In order to ‘flatten the curve’ for ICU capacity an ‘intelligent lockdown’ was promulgated half March. This intelligent lockdown was less stringent than in most other countries in the sense that people were not locked up in their houses and shops remained open (with the exception of bars and restaurants). Many shops closed voluntarily, however, encouraged by a decreased number of customers as well as relatively generous economic support measures. A visit ban was put on nursing homes in order to protect vulnerable residents. People furthermore had to keep 1.5-m physical distance and were urged to work from home (with an exception for so-called ‘essential occupations’ such as health care workers, police and people working in logistics) and sport facilities, schools and universities were closed, just like the contact occupations, museums and theatres (de Graaff *et al*., 2020; Boin *et al*., 2020*b*). In contrast to most other countries, wearing face masks was not made obligatory as the experts of the outbreak management team (OMT), led by the head of the infection prevention unit of the National Institute of Public Health (*Rijksinstituut voor Volksgezondheid en Milieu*, henceforth: RIVM) disputed their effectiveness.

The exact number of people that got infected or died because of Covid-19 during the first wave is unknown; due to scarcity of test capacity only a limited number of people has been tested during the first wave. The gap between excess mortality and registered Covid mortality was much larger in the Netherlands than in surrounding countries, also illustrating the lack of accurate data in the splintered landscape of Dutch data custodians. National death statistics however reveal an excess mortality of approximately 11,000 people during the first wave, especially among elderly persons in nursing homes (Kruse *et al*., 2020).

Policies concerning the intelligent lockdown were mainly based on hospital admission rates and behavioural models. When numbers lowered in May, restrictions were gradually lifted; nursing homes, schools and universities were (partly) reopened, just like contact facilities. Measures of physical distancing however remained in place, and workers were still urged to work from home. Economic measures to soften economic consequences of the crisis were prolonged. The government furthermore enlarged test capacity, advising people to get tested (and go in quarantine) when developing symptoms. However, enlargement of test capacity appeared to be a difficult and highly contested undertaking due to limited test capacity and lacking national coordination (see below).

**Figure 1.**
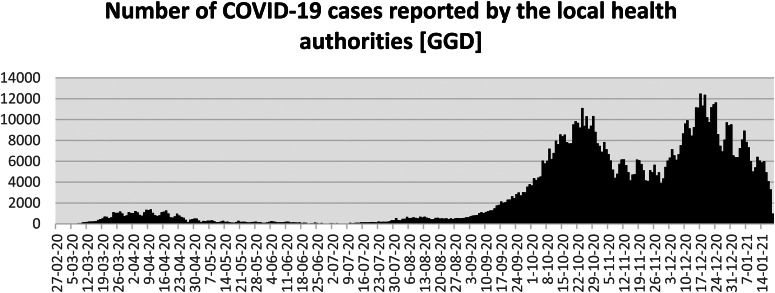
Number of COVID-19 cases reported by the local health authorities (GGD).

In the course of the summer, infection rates gradually increased again, particularly among young adults and migrant populations (see Figure 1). The central government appealed to regional authorities to take measures at the local level in order to come to a more tailored approach of combatting local outbreaks. However, after some failed attempts (i.e. the mayors of Rotterdam and Amsterdam introduced a failed experiment with the use of face masks in their city centres which was highly contested among inhabitants and local entrepreneurs), regional authorities refused and publicly declared that ‘the government should take responsibility’. When infection rates and, later on, hospitals admissions went up, restrictions were again put into place, initially with the exception of long-term care and childcare facilities, schools and sport facilities for children. It was argued that vulnerable and young people should not be isolated again. In October, and after a prolonged period of political debate, the parliament accepted an amendment to the Public Health Act to provide a more solid legal base to government's restrictions and regulations introduced in the past few months. This new law, amongst others, provided a legal ground for the physical distancing rule, fosters quarantine rules and the obligation to wear face masks in public space. Midst December, just before the Christmas holidays, after heated debates in parliament and the media and driven by a sharp increase of positive tests, hospital admission rates and infected residents in nursing homes, a new round of stringent measures was announced which put the Netherlands into a ‘full’ lockdown.

Below, we discuss how policy issues were framed in the unfolding crisis, and how this opened (and closed) policy venues for decision-making and controlling the socio-political consequences of the pandemic. Pandemic policymaking however happens against the backdrop of institutionalized national health care systems. To that purpose, we first provide a brief overview of the Dutch health care system and zoom in on the role of local and national, and public and private authorities in public health crises.

## 3. Between fragmentation and collaboration: the institutionalized context of public health crisis in the Netherlands

Dutch health care decision-making is fragmented due to its layered and decentralized nature (van de Bovenkamp *et al*., 2014). It consists of four different institutional layers: public health, social support, health care (i.e. primary care and hospital care) and long-term care. Each layer comprehends specific laws, regulations and assigned authorities. For the purpose of this analysis, we focus on public health and hospital care, and how these institutional layers have intermingled with economic-political governance.

The Public Health Act (henceforth: *Wet Publieke Gezondheid*, or WPG) stipulates authority in case of public health crisis at the national, regional and local levels. Already in January 2020, Covid-19 was classified as a category-A infectious disease. This provided the RIVM with a coordinating role and enabled regional authorities – of which there are 25 – to take measures to protect public health, like prohibiting group gatherings and closing bars, shops and sport facilities. Regional measures are worked out further in local emergency degrees. The A-classification furthermore obligates general practitioners and laboratories to report positive test results in order to foster national surveillance (Dute, 2020). The strong regional focus of most active public health measures stipulates the substantial decentralization of Dutch public health regulation in comparison with many other countries – even though, as already mentioned in the above, in practice local authorities appealed to the national government to take measures. Furthermore, public health measures like imposed quarantine policies have been hampered by national privacy regulations. The government has sought to solve this by commissioning the new ‘Corona law’ mentioned above.

Hospital care stood central in both the first and the second wave as the Covid-outbreak challenged accessibility of health care. During the first wave this particularly concerned ICU-capacity, whereas in the second wave regular hospital capacity is under pressure, also with the attempt to keep as much non-Covid care operational as possible. Governmental authority over hospital care is however weak as a consequence of the tradition of both corporatism and the reforms of regulated competition and decentralization that have characterized the development of the health care system in the past two decades (Helderman *et al*., 2005; van de Bovenkamp *et al*., 2014). Although the Dutch government has a constitutional responsibility for the efficiency, accessibility and quality of health care, it is not equipped to accomplish these responsibilities under its own strength. Instead, the health care system has been built upon corporatist arrangements whereby the state has delegated important responsibilities to the various associations of providers and health insurers (Helderman *et al*., 2005). As a consequence, the government and health care providers experience a high degree of public–private dependency and health care policies are highly negotiated.

The introduction of regulated competition in the mid-2000s has established a system in which hospitals compete (both in terms of quality of care and costs) for a contract with health insurers, and health insurers in their turn compete for the insured (Maarse *et al*., 2015). The system is however highly regulated: the Dutch Health care Authority (*Nederlandse Zorgautoriteit*, henceforth: NZa) has a central role in setting the conditions for regulation and hospital reimbursement to protect the public good (van Erp *et al*., 2020). Professional associations and other interest groups are however highly involved in decision-making – revealing the path dependent development of the Dutch health care system and its reliance on negotiation and trust. This regulatory capacity and negotiated order of the health care system is also visible in the Covid crisis: the NZa deliberately adjusted the conditions for reimbursement (e.g. allowing electronic consults for full reimbursement), negotiated with the national association of health insurers about the reimbursement of Covid care (which was, naturally, not included in the 2019 negotiations underlying the reimbursement agreements of 2020), and attempted to coordinate the accessibility to regular (i.e. non-Covid) care when urgent regular care provision (e.g. cancer treatment) was supplanted by Covid-care.

At a regional level, finally, the system of regulated competition is substituted with a collaborative institutional infrastructure for acute care. Hospitals collaborate within Regional Consultative Bodies for Acute Care (*Regionaal Overleg Acute Zorg*, ROAZ) for the coordination of acute care delivery and also have a coordinating role in times of incidents or crisis, for instance about the distribution of patients and bed capacity. There are 11 acute care regions who collaborate in a national network (*Landelijk Netwerk Acute Zorg*, henceforth: LNAZ) to coordinate patient care. In the following, we will further discuss the importance of this developing institutional crisis infrastructure for the framing of the Covid-crisis and how policy-decision making ensued. Rather than a chronological account of the Dutch response to Covid-19, we examine three topics that were key to the national debate and that enable us to analyse the resilience of the Dutch health care system; that is, its ability to cope with unanticipated dangers and to ‘bounce back’ (Wildavsky, 1988).

## 4. Aligning hospital care^2^

In the Dutch health care system, hospitals do not have an institutionalized public role in times of health crisis. Hospitals have their own emergency plans and are loosely organized in regional and national acute care networks. Individual crisis management however soon appeared insufficient during the Covid crisis. Hospitals were confronted with a large increase of severely ill patients, many of them needing ICU care. Regular care was scaled down to accommodate the growing group of Covid-patients. In the second week of March, hospitals in the South started to get overwhelmed (which was also due to the institutionalized low bed capacity, see below). Physicians contacted colleagues in Northern hospitals to take over patients. This mechanism however soon failed; too many patients were in need of immediate medical support and intensive care treatment. This was the point that a new acute care infrastructure was set up, initiated by LNAZ who did not have a decisive role before. In close contact with the Ministry of Health, it created a centre for the (re)distribution of patients, supported by the Dutch army (*Landelijk Centrum Patiënten Spreiding*, henceforth: LCPS). This more or less happened overnight. The LCPS took over the distribution of patients. Hospitals were required to provide their admittance rates twice a day, and if needed patients were transferred to other hospitals ‘with empty beds’. When the curve flattened early summer, the LCPS was dismantled. It got back into force half October when hospitals faced a second wave of patients and regular care was again scaled down. However, this time the focus was much more on an equal distribution (‘fair share’) of patients in order to keep up with regular care provision as much as possible. The LCPS, that had quickly grown out from an improvised call centre into a well-equipped and well-organized agency, was provided with the mandate to force unwilling hospitals to accept Covid-patients, showing the increased centralization of decision-making, albeit coordinated through the LNAZ and thus largely under the control of the acute care regions. For example, the LNAZ, in close contact with the medical associations, set criteria for which patients could be transferred.

Hospitals publicly worried about the financial consequences of expensive Covid care provision (i.e. patients were often transferred to ICU departments, usually for several weeks) for which no financial agreements existed, and the loss of regular care on which their financial position was based. Moreover, they were afraid to lose patient groups to competitive hospitals if they would turn into ‘Covid-hospitals’. Hospitals anxiously looked at neighbouring hospitals and whether they were scaling down or up and might ‘steal away patients’ (as a manager of a university hospital explained in an interview). Also, when some hospitals started to use their private clinics to offer regular care, this was seen as a breach of the gentlemen's agreement of ‘being in this together’. The same goes for ICU capacity. As this has largely been centralized in Dutch health care (see below) the Covid crisis opened up possibilities for smaller hospitals to increase their ICU capacity, thereby also strengthening their acute care provision. Such competitive logics had to be negotiated constantly in ROAZ and LNAZ contexts. Disputes over extra costs were however mostly settled when health insurers offered generous settlements. Moreover, larger hospitals – mostly university medical centres – have generally gained more in ICU capacity than smaller ones.

The need to ‘act as a whole’, which fits in with the public health crisis logic, and the private interests to safeguard a hospital's competitive position following the logic of the health care market, exemplifies the clash of two institutional layers of the Dutch health care system; that of the regulated market and that of the crisis organization. However, and especially in the first weeks, old traditions of collective action relived as national agencies, the acute care network, health insurers and hospitals tactfully drew up new collective infrastructures like the LCPS, while the NZa and the health insurance agency negotiated about reimbursement rules to keep Covid care going and soften the pressure of market competition. The NZa negotiated the tariffs and conditions for reimbursement, as well as the compensation of financial losses due to the scaling down of regular care provision. Similar initiatives were taken to compensate primary care providers (e.g. dentists and physiotherapists) for the sudden decline in their service delivery. Also, reimbursement rules were adjusted to enable alternative care provision like e-health and Covid after-care.

## 5. Negotiating scarcity

The first months of the Covid-crisis were dominated by discussions on scarcity and collective attempts to meet urgent needs. Scarcity was multiple; a shortage of ICU-capacity (especially ICU-beds and ventilators), specialized workforce, personal protective equipment (PPE) and test capacity. Scarcity was both due to the pandemic (i.e. a worldwide unmatched demand for PPE, medicines and ventilators) and institutionalized features of the Dutch health care system, like a limited number of ICU-beds and restricted test capacity.

The scarcity problems were widely covered in the media, and the Minister of Health regularly had to report to parliament about the shortages and solutions to solve these. With regards to PPE, a problem that was particularly threatening (and frightening, interviewees stressed) in the first weeks, the Ministry of Health took up a central coordinating role. Together with the LNAZ, PPEs were imported from all over the word – although the media also reported on costly and failed attempt to get hold on batches of face masks – and distributed among hospitals (nursing homes were only included later on). The government not only coordinated the purchase of equipment, but also took care of its finance, which conflicts with the institutionalized private responsibility of hospitals, but also illustrates the central approach of the first months.

Another main problem in the first phase was the shortage of ICU-beds and ventilators. Compared to other OECD countries, the Netherlands has a low number of hospital beds and particularly ICU-beds (NVZ, 2018). It was calculated that ICU capacity had to be doubled to accommodate all Covid-patients in need of intensive care treatment; expanding capacity from 1150 to 2400 beds – a number that figured prominently in the media during the first crisis (de Graaff *et al*., 2020). ICU-beds are part of the acute care infrastructure, which is regulated by the Ministry of Health and the NZa. Regulations are based on both costs and quality and have led to a centralization of ICU care as well as an overall decrease of ICU capacity. As a consequence, not every hospital has a ‘level 1’ ICU department that can provide artificial ventilation, which was needed in case of severe Covid-related sickness. Some patients were sent to Germany that traditionally has a much higher ICU capacity. Scarcity of ICU care was also due to the long-lasting problem of shortage of specialized nurses. This impeded a quick upsurge of ICU-capacity. Hospitals found solutions in speed-training nurses; un-qualified registered nurses were trained to assist ICU-nurses in taking care of Covid-patients. The Health care Inspectorate adjusted ICU-norms quality rules to allow for this ad-hoc solution. Furthermore, the Inspectorate enabled unlicensed nurses to return to their former jobs in hospitals and nursing homes. These examples illustrate again the logic of tact in which public and private actors collectively figured out solutions to the problems they were confronted with, each within their own roles but also with a sense for collective needs.

Testing for Covid-19, a central element in international policies, however appeared an even more complicated and conflictuous undertaking that got more and more politicized. Scarcity of tests and laboratory capacity was due to both a worldwide high demand for tests, and an institutionalized restricted and highly regulated use of laboratory capacity for public health. In the Netherlands, only a few laboratories have been certified to conduct tests in case of public health problems. It became apparent that Dutch microbiologists had been successful in restricting lab capacity, arguing that highly specialized knowledge is needed and cannot be offered by private services (Stokmans *et al*., 2020). Furthermore, testing itself has been assigned to the local health authorities that work for municipalities and lack a national executive coordinating structure which made it difficult to quickly upsurge capacity to test a large number of citizens. This fragmented infrastructure meant that coordination of tests was difficult and that capacity was way too low. Experts (and politicians) argued that testing should be central to the crisis strategy, which was repeatedly expressed within the media and supported by employer organizations. The importance of testing was further underscored by the obligation after the summer to ‘stay at home and get tested’ in case of health complaints. Schools and health care organizations increasingly faced shortage of personnel as employees had to stay at home awaiting test results, which took days or even a full week due to long waiting times for testing and insufficient lab capacity. The Minister of Health had to testify to parliament and the media about the system failure repeatedly, yet also (and increasingly) articulated his own frustration about the field's incompetency of ‘making it work’ despite the huge monetary investments. In an interview in a national newspaper early December, the Minister of Health, Hugo de Jonge, stated that ‘We have been improvising throughout the crisis’ (Aharouay and van den Dool, 2020) – revealing his annoyance with failing institutional arrangements and lack of institutionalized hierarchical power in the health care system.

Historically grown vital health care infrastructures such as ICU and test capacity thus proved to be a major issue during the crisis and in a way also prevented its solution. Health care reforms of the past few years to curb rising costs have made the system ‘leaner’, yet also appeared insufficient to ramp up capacity in case of crisis. Although ICU capacity was taken up quickly during the first wave, test capacity took longer to organize and was only realized halfway through the second wave and remained contested. The logics of appropriateness and tact, that had been quite successful in the first months to overcome the fragmented health care system, were replaced by a more hierarchical-bureaucratic logic of decision-making as both the media and field actors (i.e. mayors, hospitals and employer organizations) expected control and leadership from the central government.

## 6. Experts and expertise: a solid and contested policy basis

The reliance on experts is institutionalized in the Public Health Act which provides a leading role to the RIVM and the Health Council (*Gezondheidsraad*) in case of public health crisis. The RIVM closely monitors infection and hospital admission rates and uses data models to analyse the impact of policy measures on the spread of the virus. These models have become key instruments for policy decision-making. The Health Council advises the Ministry of Health (amongst others) about vaccination policies and ethical issues. The role of experts and scientific knowledge is furthermore anchored in the national OMT that advises the cabinet about policy measures. The OMT is chaired by the director of the infection prevention unit at the RIVM and includes various scientific experts; all medical doctors, epidemiologists and virologists. This rather narrow focus on medical-epidemiological knowledge has been criticized, however, especially when the societal impact of Corona measures became clear (e.g. loneliness among elderly and children in unsafe home situations that could no longer go to school or day care because of the lock down). Social and economic scientists were hardly involved at the start, nor was the ‘normal’ advisory structure of the state, which however responded by setting up collaborative projects in the context of the Socioeconomic Council. Also, the RIVM set up a behavioural unit, making use of experts across the country. Social science input into decision-making however remained small.

The OMT has played a crucial role in governmental decision-making. Initially, the cabinet (and the OMT) acted reluctantly in issuing rules, which also resulted in the initial ‘intelligent’ lock down instead of a ‘full’ one. They argued that schools should remain open. As children were not the main sources of contamination, they should keep their social lives, and parents should be able to work from home. This more careful approach was soon thwarted by the medical specialist association that argued that not closing schools immediately would be irresponsible. Referring to ‘the Italian situation’ they appealed to broader worries among teachers and parents about safety at school. During a press conference of Prime Minister Rutte 2 days later, it was indeed announced that all day care, schools and universities would close that same week. This occurrence also illustrates the role of the Dutch media in national policy-making, which has sharply increased in the past few months. The media has had a striking role in the sharpening of restrictions as ample room was given to critical experts striving for more stringent measures, yet the media was much more reluctant to express criticism on restrictions.

In the course of the summer a more contested and dispersed picture emerged. Infection and mortality rates and hospital admissions had sharply decreased, and restrictions were lifted. Mayors and other local authorities, at the same time, increasingly felt responsible to keep transmission rates low while also recognizing that people were out more often and violated the Corona-rules (e.g. keeping 1.5 m distance). Local authorities and (self-announced) experts and public figures increasingly started to criticize policy measures and national political leaders in talk shows, on social media and in the newspapers. Striking example is the so-called ‘Red Team’ consisting of a wide variety of scientists, (retired) physicians and opinion makers who argued for a much more stringent policy approach like the obligation to wear face masks and to close schools in case of infections. These ‘critical actors’ actively approached mayors, policy-makers from the Ministry of Health and journalists, pointing at more stringent policies in other countries that might be more successful in curbing the virus. They got support from (amongst others) associations of teachers and also some experts from the OMT openly supported these ideas. As a result, a new policy venue for a more ‘law-and-order’ approach was created that not only conflicted with the more moderate policy approach of the first wave, but also embarked on the ‘tactful’ approach of collective action and decision-making in the first phase of the crisis. This law-and-order approach was played out full midst December when infection rates went up and hospital and nursing home directors warned for an overwhelmed health care system with Christmas parties ahead. Prompted by an OMT advise, as well as publicly expressed worries of the mayors of the safety regions who did not manage to keep shopping areas quiet and signalled many violations of the 1.5 m distance norm, the government issued an immediate and full lock down. Although most citizens were caught by surprise, opinion surveys revealed that the measures were supported by a majority of the population.

Experts and ‘science’ play a central role in Dutch crisis policy making. Covid-19 policies are presented as evidence-based (following the logic of consequence), although the government and especially Prime-Minister Mark Rutte have frequently underscored the deep uncertainty in which decisions are taken, also revealing the ‘probe and learn strategy’ of the government (Ansell and Boin, 2019). The politization of the crisis, which has become increasingly apparent after the summer, however left little room for such a pragmatist approach. Experts who plead for more stringent measures have become more dominant, calling on a ‘politics of urgency’ (Versluis *et al*., 2019) that is supported by most media. This has given room for a more hierarchical and bureaucratic logic of decision-making, allowing for strict lock down measures at the time of writing. Shops, contact vocations, day care, schools and universities are closed, and traveling outside the country has been all but forbidden – yet nursing home and mental care facilities have remained open as ‘locking up’ older persons and vulnerable people is considered unethical.

## 7. Discussion

Public health crises, like the Covid-19 outbreak, require decisive intervention at both national and local levels. How such interventions are structured and play out in policy and health care practice depends on the institutional infrastructure of health care systems, revealing its institutionalized resilience capacity (Ansell *et al*., 2016). In the unfolding Covid crisis in the Netherlands, crisis management and decision-making were initially led by March and Olsen's (1989) logics of consequence and appropriateness; authoritative agents were largely capable of effective and legitimate decision-making. The Ministry of Health and the national acute network took over the purchase of necessary equipment and supported the expansion of ICU-capacity through the negotiation of both quality and reimbursement regulations. At the same time, socio-economic measures were taken to curb the virus outbreak and soften societal consequences, and local authorities participated in executing national safety measures. We have shown how ‘tact’ was used to align the much more top-down orientation of the public health infrastructure with the decentralized and fragmented structure of the hospital market to deal with this situation in which traditional scripts and cognitive schemata had fallen short (Kornberger *et al*., 2019). As long as the deployment of tact (‘connecting thoughts to actions’) resulted in workable interventions, this approach indeed strengthened the resilience of the system. Yet, when partisan interests became too heterogeneous and the various thoughts underlying these interests no longer resulted in unitary decisions and actions, decisive interventions became subject to the logic of bureaucratic/governmental political decision-making in which decision-makers no longer attempt to defend a united decision-making strategy. This indeed happened when the acute crisis turned into a more enduring one, and ‘cracks and gaps’ emerged in the policed policy strategy, fostered by the intervention of more conservative experts that got a voice through the media that was on top of all details of the Covid-crisis. The lacking testing infrastructure further politized crisis management, also revealing the downsizes of a fragmented and lean-oriented health care system. Fierce and widespread criticism, both from members of the OMT and ‘outside’ experts that have grown into TV personalities as the crisis prolonged, as well as increasing infection rates, have engendered stringent lockdown measures that had seemed impossible in the first months of the crisis. With the 2021 elections ahead, this means an additional test of the resilience of the Dutch socio-political system and the Dutch health care system. Covid-19 not only unmasks some of the most critical features of the Dutch health care system, it has also become the litmus test for political decision-making in times of crisis.

## Footnotes

1 In this section, we make use of the Hepl-blogs we published in the course of 2020, see https://www.cambridge.org/core/blog/2020/04/06/the-netherlands-response-to-the-coronavirus-pandemic/.

2 In this section, we make use of our empirical research (observations, interviews and document analysis) on the Covid outbreak and decision-making structures in the Netherlands, see (Bal *et al*., 2020; Wallenburg *et al*., 2020).
